# One-Year Changes in Activities of Daily Living, Usability, Falls and Concerns about Falling, and Self-Rated Health for Different Housing Adaptation Client Profiles

**DOI:** 10.3390/ijerph18189704

**Published:** 2021-09-15

**Authors:** Agneta Malmgren Fänge, Carlos Chiatti, Anna Axmon

**Affiliations:** 1 Department of Health Sciences, Faculty of Medicine, Lund University, SE-221 00 Lund, Sweden; 2 Tech4Care Srl, Falconara Marittima, 60015 Ancona, Italy; c.chiatti@tech4care.it; 3 EPI@LUND, Division of Occupational and Environmental Medicine, Lund University, SE-221 00 Lund, Sweden; anna.axmon@med.lu.se

**Keywords:** home modification, disability, clusters, longitudinal, health, intervention, prioritization, evaluation

## Abstract

The purpose of this study was to investigate one-year changes and differences in changes in activities of daily living (ADL), usability, a history of falls, concerns about falling, and self-rated health across five housing adaptation (HA) client profiles identified previously using a cluster analysis approach: older adults with low level of disability (*n* = 59); older adults with medium/high level of disability (*n* = 26); adults with low level of disability (*n* = 10); adults with high level of disability (*n* = 8); and older adults with medium level of disability including at least moderate cognitive impairment (*n* = 5). Comparisons between the five profiles include secondary analyses aggregating those with low level of disability and those with medium/high level of disability. Changes within the client profiles demonstrate a complex pattern of improvements and declines, depending on outcome, with no profile showing consistent improvement or decline across all outcomes. The risks of deterioration over one year were the highest among those with cognitive impairments at baseline, but no recommendation of prioritization decisions based on baseline profiles can be made. Instead, it seems that all HA clients, independently of baseline profile, are at risk of increasing disability over time and require follow-up evaluations regularly.

## 1. Introduction

Global population trends indicate that the group of people 80+ years is the most rapidly growing age group, expected to triple between 2019 and 2050 [1]. In parallel, aging-in-place policies are being promoted worldwide as a better alternative than institutional care [2,3]. Since age is related to disability [4], this will lead to more people with disability living in their own homes. Accordingly, this demographic development challenges society to come up with efficient and effective provision of home-based interventions to counteract disability and promote health and wellbeing.

Disability is an umbrella term for impairments, activity limitations, and participation restrictions, i.e., the negative aspects of the interaction between a person with a health condition and the person’s context [5]. Thus, one way to counteract disability might be to change the context [6], for example by adapting the home environment. 

A common environmental intervention is a housing adaptation (HA), defined as a structural modification of the physical home environment [7,8]. In Sweden, after needs assessment, people can receive a grant covering the full costs for a HA from the municipality, with the aim of enhancing independent living in one’s own home [9]. The most common adaptations are removal of thresholds and installation of grab bars or stove guards. The population receiving HA differs in health as well as in standards and types of housing they live in. Although 75% of them are older than 70 years [10], and the majority are facing age-related health decline and dependence due to barriers in the housing [11], younger people with acute or progressive diseases or injuries also receive HA [12]. Most HA clients receive health and social care in parallel, e.g., provision of mobility devices and assistance with activities of daily living (ADL) [12,13,14]. Previous research has shown that HA has a positive effect on activity, such as reduced dependence in ADL and increased activity performance [8,15,16], usability in the home [8], improved fall-related outcomes [17,18], and self-rated health [19,20]. Hence, it seems that a HA can counteract disability.

From a societal perspective, there is an increasing need of providing evidence-based and effective health care interventions, including follow-up schemes, to improve patient outcome and to ensure that finite health resources are used wisely [21]. Even though in Sweden, a HA is not considered a health care intervention, given the strong relationship between health conditions and context for the development of disability, research-based guidance on follow-up schemes after HA may have the potential to capture those clients in need of close monitoring to prevent disability. Follow-up schemes are increasingly relevant, as they can support the evaluation of mid- and long-term effects of interventions, ultimately clarifying their efficiency and cost-effectiveness [22]. In a previous mixed methods study [23], five HA client profiles characterized by age and level of disability, based on ADL dependence, usability in the home, number of functional limitations, cognitive impairment, history of falls, concerns about falling, and self-rated health, were identified and validated (older adults with low level of disability; older adults with medium/high level of disability; adults with low level of disability; adults of high level of disability; and older adults with medium level of disability). 

The possibility to identify profiles of clients, created through the identification of intragroup similarities and intergroup differences, can have a twofold benefit. From the perspectives of health care and social service providers, knowledge about the characteristics of the client population would be useful for the allocation of available resources to those at risk of increasing disability, as well as for the development of research-based strategies for different interventions in the home, not restricted to HA. From the perspective of the care professionals, these client profiles may provide a better understanding of how home-based interventions could be delivered more effectively to specific groups of HA clients based on their different characteristics. In addition, care professionals could use client profiles to differentiate between groups of clients based on their expected development trajectories over time. For example, knowledge about client profile trajectories can facilitate client-centered decisions on follow-up schemes as well as implementation of other interventions. Accordingly, the aim of this study was to investigate one-year changes in ADL, usability in the home, a history of falls, concerns about falling, and self-rated health across different HA client profiles. A further aim was to investigate differences in changes over one-year between the client profiles.

## 2. Materials and Methods

### 2.1. Study Context and Design

This longitudinal study uses data from the ResHA study [7], a controlled trial involving people applying for a HA grant in three south Swedish municipalities. 

### 2.2. Data Collection 

The participants were identified and enrolled by the municipality occupational therapists conducting the HA needs assessments. Data were collected by occupational therapists during home visits, using validated instruments as well as study-specific questions [7]. Data collection was performed at the same four time points: at T1 (max. 1 month before the start of the HA) and 3, 6, and 12 months after the HA was finalized (T2, T3, and T4, respectively). For this study, data collected at T1 and T4 were used. 

#### 2.2.1. Study Population 

Individuals aged 20 years or more and applying for a HA grant were eligible for inclusion. Excluded were those living in sheltered housing, and those not being able to read or speak Swedish. Baseline data were collected between January 2013 and December 2015 (T1), in total *N* = 241. After one year (T4), *n* = 116 participants were available for follow-up [8]. Only clients with complete information on all variables needed to place in one of the client profiles were included in the analyses for this study (*n* = 108). 

#### 2.2.2. Outcomes

We investigated one-year changes in health-related outcomes, i.e., ADL dependence, usability in the home, concerns about falling, self-rated health, and history of falls among previously identified profiles of HA clients [23]. 

ADL dependence was assessed using the ADL staircase, extended to allow the response for each item to be either independent without difficulties, independent with difficulties, partly dependent, or dependent [24]. The response options were coded as 0, 1, 2, and 3, respectively, with higher scores indicating more dependence. Sum scores were calculated for instrumental ADL (I-ADL; cooking, transportation, shopping, and cleaning), personal ADL (P-ADL; feeding, transfer, using the toilet, dressing, and bathing), and total ADL (all nine items) when values were available for all items included. 

Usability in the home, i.e., the person’s perception of the extent to which the home environment supports different activities, was measured using the Usability in My Home (UIMH) instrument, revised version. Sum scores were calculated for three components of usability: self-care (going to the toilet, personal hygiene, preparing meals, preparing snacks, and moving around the home with or without a mobility device), social (socializing with family and friends in the home, contacting others via telephone or Skype, and watching TV or listening to the radio), and outdoor leisure (entering the house, picking up the mail, and engaging in hobbies and leisure activities in the home). Total scores were 0–25 for self-care and 0–15 for social and leisure/outdoor aspects, respectively [25]. Higher scores indicate better usability of the home. Concerns about falling were measured by the short form of the Falls Efficacy Scale-International (FES-I) [26]. This instrument includes seven activities (getting dressed or undressed; taking a bath or shower; getting in or out of a chair; going up or down stairs; reaching for something above your head or on the ground; walking up or down a slope; going out to a social event), each assessed on a four-point scale (1–4), adding up to a total score (7–28) with a higher score indicating more concern. Sum scores were calculated for those with missing values on no more than one item, with those with only valid values for six items (i.e., one missing) being assigned the mean value of those six items multiplied by seven and rounded up to the nearest integer. 

Self-rated health was assessed by means of the Euro-QoL 5D Visual Analogue Scale (EQ-VAS) [27]. Respondents were asked to rate their overall health on the day of the interview on the vertical visual analogue scale, from 0 to 100, where 0 indicates the worst and 100 the best imaginable health. The EQ-VAS has been tested for validity and reliability [27,28].

A history of falls during the last six months was assessed by collecting the self-reported number of retrospective falls. The study-specific questions: “Have you fallen in the last six months” (Yes/No), “Have you nearly fallen in the last six months” (Yes/No), and “If yes, how many times?” (asked for both falls and near falls) were used. 

### 2.3. Client Profiles

Analyses for this study were based on HA client profiles initially defined based on quantitative data, applying cluster analysis methodology [29]. The profiles were further refined by means of a mixed methods approach combining quantitative data with data from individual interviews, collected at baseline (T1) [23]. The client profiles were identified based on age and level of disability according to the International Classification of Functioning, Disability and Health, ICF [5]. The level of disability is relative and not based on cut-off values. Instead, it was identified based on comparisons between the profiles, according to cluster analysis procedures. The qualitative data were used to corroborate the five client profiles, showing the following characteristics (Table 1). 

For a description of the study population, see Table 2. 

### 2.4. Data Analysis 

Analyses were performed including those with data from both T1 and T4, as well as complete data for categorization at T1 (see also Section 2.2.1; *n* = 108).

In the main analyses, comparisons between the five client profiles were made. However, due to the smaller size of profiles 3–5, we also performed secondary analyses comparing profiles 1 and 3 (i.e., those with low level of disability) with profiles 2 and 4 (i.e., those with medium/high level of disability). 

Changes in continuous variables (ADL sum scores, usability in the home components, EQ-VAS, FES-I) were calculated by subtracting the value at T1 from the value at T4, i.e., positive changes (>0) indicate an increase in the variable from T1 to T4. Thus, an increase in the usability in the home components and EQ-VAS represents favorable outcomes (improvement), whereas an increase in the ADL sum score and FES-I corresponds to unfavorable outcomes (decline). The changes were assessed by P-P-plots. All three measures of ADL as well as all three components of usability, self-rated health (EQ-VAS), and concerns about falling (FES-I) were considered normally distributed, and thus the overall differences between clusters were analyzed using analysis of variance (ANOVA). In the main analyses (i.e., when comparing all five profiles), post hoc tests for pairwise comparisons were performed using the least significant difference *t*-test (LSD). ANOVA was performed both unadjusted (i.e., with only the profile included as independent variable) and adjusted for potential confounders (i.e., including functional limitation and cognitive function in the model). Overall worsening of fall history was defined as (1) not having fallen at T1 but at T4, or (2) having fallen at both T1 and T4, but more times at T4, or (3) not having fallen or nearly fallen at T1 but having nearly fallen at T4, or (4) not having fallen at either T1 or T4 but having nearly fallen at both T1 and T4 with more times at T4. Non-worsening (i.e., improvement or no change) was defined as not fulfilling the criteria for worsening while having complete information on all four variables. Differences in worsening of fall history were analyzed using logistic regression, estimating odds ratios (ORs) with 95% confidence intervals (CIs) for comparisons with client profile 1 in the main analyses and client profiles 1 and 3 combined in the secondary analyses.

#### Potential Confounding

We considered baseline functional limitations and cognitive function as potential confounders in the analyses. Functional limitation was determined using 12 items from the personal component in the Housing Enabler Instrument [30] measured as present (=1) or absent (=0). A total score 0–12 was calculated; the higher the score the larger the number of limitations. The Montreal Cognitive Assessment (MoCA) [31] was used to assess different aspects of cognitive function on a 0–30-point scale. A score of 25 points or below is considered to indicate cognitive impairment. The total score for the baseline MoCA test was used in the adjusted models.

### 2.5. Ethics

The study was approved by the Regional Ethical Review Board in Lund (dnr 2012/566) and conducted in accordance with the Declaration of Helsinki [31]. Participation in the study was voluntary and written informed consent for inclusion was given by the participants prior to the data collection. All data were collected at home visits, which requires significant sensitivity to the participant’s life situation. The population studied is known to include a high number of individuals who could be described as frail and who might have cognitive impairments. All data collectors had extensive professional experiences from home visits to people with disabilities, and they had also specific training in collecting research data in this context. All data are deidentified and stored digitally in encrypted format. Paper versions are stored in locked areas to which only the researchers have access. 

## 3. Results

The number of people included in the different analyses are presented by client profile in Table 3 and Table 4, respectively.

### 3.1. Changes in Outcomes Per Client Profile 

**Figure 1:**
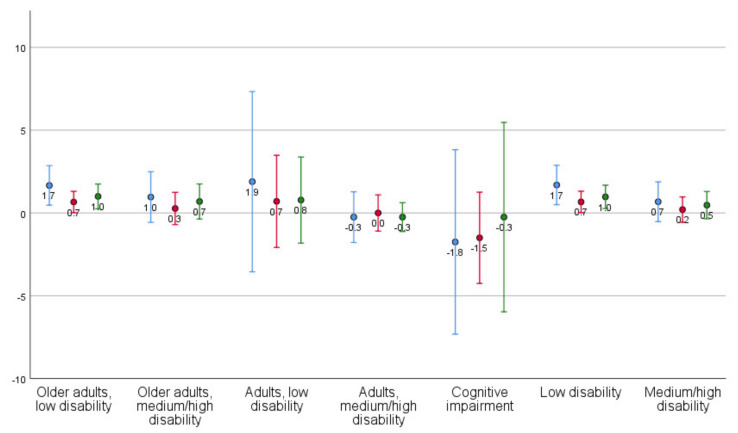
Mean (dots) changes with standard deviation (lines) in ADL dependence, per client profile and aggregated client profiles (blue line = total ADL, red line = P-ADL, green line = I-ADL). Values > 0 indicate decline (i.e., increased dependency) from T1 to T4.

**Figure 2:**
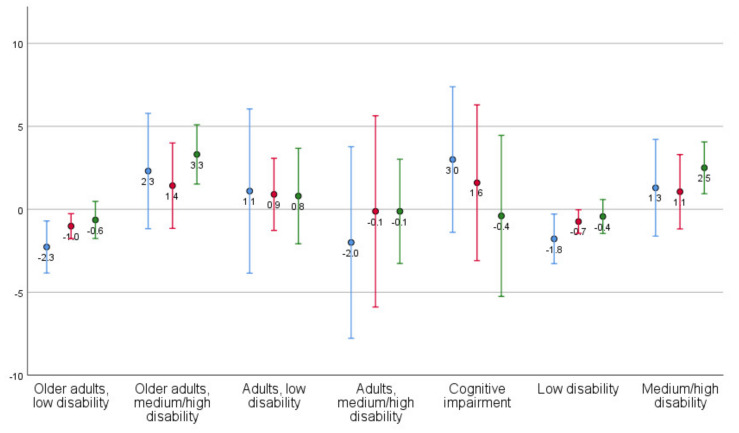
Mean changes (dots) with standard deviation (lines) in Usability in the home, per client profile and aggregated client profiles (blue line = self-care, red line = social, green line = outdoor leisure). Values > 0 indicates improvement (i.e., higher usability) from T1 to T4.

**Figure 3:**
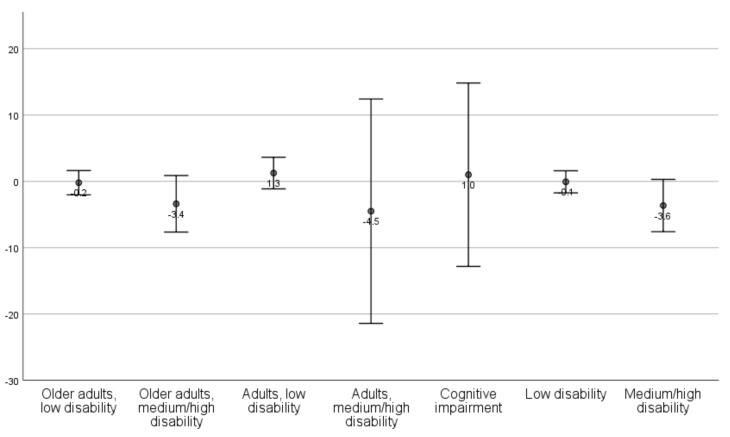
Mean changes (dots) with standard deviation (lines) in concerns about falling, per client profile and aggregated client profiles. Values > 0 indicates decline (i.e., increased concern about falling) from T1 to T4.

**Figure 4:**
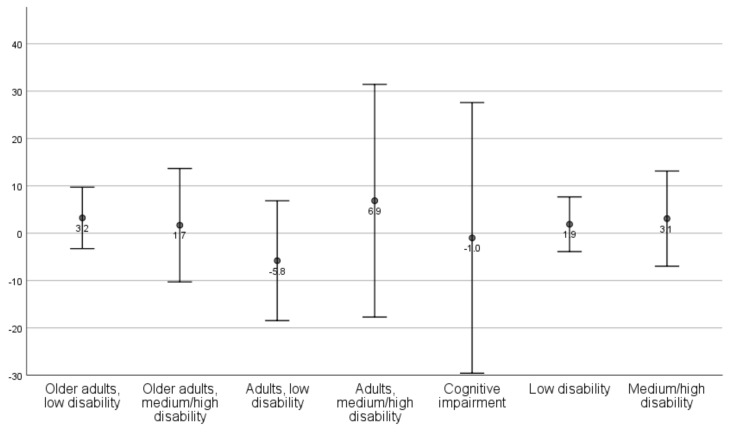
Mean changes (dots) with standard deviation (lines) in self-rated health, per client profile and aggregated client profiles. Values > 0 indicates improvement (i.e., increased self-rated health) from T1 to T4.

Mean changes with standard deviations are presented by client profile (Table 3; Figure 1, Figure 2, Figure 3 and Figure 4) and aggregated client profiles (Table 4; Figure 1, Figure 2, Figure 3 and Figure 4). Older adults with low level of disability became less concerned about falling and had improved self-rated health during the year. However, they rated the usability of the home as worse and showed increased dependency in ADL. Older adults with medium/high level of disability demonstrated increased ADL dependence, but rated the usability of the home and self-rated health to be better, and were less concerned about falling. Adults with low level of disability showed less ADL dependence and better usability, but had more concerns about falling and a decline in their self-rated health. Adults with high level of disability were stable or improved in ADL independence, became less concerned about falling over one year, and demonstrated improvement in self-rated health. They did, however, rate the usability of the home worse. Older adults with medium level of disability including at least moderate cognitive impairment improved in ADL independency as well as the self-care and social aspects of usability but demonstrated a decline in the outdoor leisure aspect as well as in concern of falling and self-rated health. 

**Table 3:** Values at T1 and T4, and one-year-change for the five original client profiles with *p*-values for comparisons of all groups estimated using analysis of variance (ANOVA). Favorable outcomes (i.e., less dependence in ADL, increased usability and self-rated health, and less concerns about falling) are marked with grey shading.

	Older Adults, Low Disability			Older Adults, Medium/High Disability			Adults, Low Disability			Adults, Medium/High Disability			Cognitive Impairment			*p*-Value
	*n*	M	SD	*n*	M	SD	*n*	M	SD	*n*	M	SD	*n*	M	SD	Unadj/Adj ^1^
**ADL**																
T1	59	9.8	5.7	26	12.9	6.2	10	7.7	3.5	8	19.5	3.4	5	8.2	5.0	
T4	59	11.5	6.3	26	13.8	5.5	9	9.2	6.7	8	19.3	4.7	4	4.8	4.9	0.488/0.677
Change	59	1.7	4.6	26	1.0	3.8	9	1.9	7.1	8	−0.2	1.8	4	−1.7	3.5	
**P-ADL**																
T1	59	3.3	3.0	26	4.5	3.4	10	2.3	1.8	8	8.6	2.8	5	3.2	2.3	
T4	59	3.9	3.5	26	4.8	3.8	10	3.0	4.1	8	8.6	3.5	4	1.0	0.8	0.522/0.284
Change	59	0.7	2.5	26	0.3	2.4	10	0.7	3.9	8	0.0	1.3	4	−1.5	1.7	
**I-ADL**																
T1	59	6.5	3.3	26	8.4	3.3	10	5.4	2.1	8	10.9	1.0	5	5.0	3.1	
T4	59	7.5	3.4	26	9.1	2.2	9	5.9	2.7	8	10.6	1.6	4	3.7	4.1	0.733/0.970
Change	59	1.0	2.9	26	0.7	2.6	9	0.8	3.4	8	−0.2	1.0	4	−0.2	3.6	
**Usability: Self-Care ^2^**																
T1	59	21.0	3.6	26	12.3	6.2	10	18.0	4.5	8	11.5	4.6	5	17.0	3.2	
T4	59	18.8	6.0	26	14.7	6.3	10	19.1	4.8	8	9.5	6.6	5	20.0	1.2	0.037/0.072
Change	59	−2.3	6.0	26	2.3	8.6	10	1.1	6.9	8	−2.0	6.9	5	3.0	3.5	
**Usability: Social ^3^**																
T1	59	13.6	1.4	26	9.4	4.5	10	13.0	2.7	8	10.8	3.7	5	10.0	3.5	
T4	59	12.6	2.8	26	10.8	3.6	10	13.9	1.4	8	10.6	5.3	5	11.6	0.9	0.144/0.340
Change	59	−1.0	2.9	26	1.4	6.4	10	0.9	3.0	8	−0.1	6.9	5	1.6	3.8	
**Usability: Outdoor Leisure ^4^**																
T1	59	9.8	3.2	26	4.3	3.0	10	9.1	1.4	8	6.3	3.2	5	11.0	2.6	
T4	59	9.1	4.3	26	7.6	4.0	10	9.9	3.6	8	6.1	3.8	5	10.6	2.7	0.005/0.222
Change	59	−0.6	4.3	26	3.3	4.4	10	0.8	4.0	8	−0.1	3.8	5	−0.4	3.9	
**Concerns about Falling**																
T1	48	14.3	4.4	18	18.6	5.3	9	18.7	5.8	8	18.6	8.3	3	12.7	6.0	
T4	46	13.9	4.8	18	14.9	5.2	4	18.3	7.8	4	14.0	7.3	5	15.6	4.7	0.330/0.200
Change	39	−0.2	5.6	13	−3.4	7.1	4	1.3	1.5	4	−4.5	10.6	3	1.0	5.6	
**Self-Rated Health**																
T1	59	61.7	18.5	22	51.0	19.6	10	59.8	18.1	8	42.5	22.7	5	55.0	14.1	
T4	57	65.0	20.1	22	57.3	22.6	10	54.0	22.9	7	48.3	26.6	5	54.0	32.1	0.814/0.554
Change	57	3.2	24.5	19	1.7	24.8	10	−5.8	17.7	7	6.9	26.6	5	−1.0	23.0	

^1^ Adjusted for functional limitation and cognitive function at baseline *p*-values for statistically significant post hoc test (least significant difference *t*-test) in unadjusted analyses: ^2^ Profile 1 vs. 2, *p* = 0.005; ^3^ Profile 1 vs. 2, *p* = 0.020; ^4^ Profile 1 vs. 2, *p* = 0.001; profile 2 vs. 4, *p* = 0.049.

When aggregating the client profiles into low disability and medium/high disability, respectively, those with low disability demonstrated improvements in self-rated health and had less concerns about falling but declined in ADL and all aspects of usability. Those having medium/high disability demonstrated improvements in self-rated health and usability and became less concerned about falling. However, they displayed increased dependency in ADL (Table 4). 

**Table 4:** Values at T1 and T4, and one-year-changes for the aggregated client profiles with *p*-values for comparisons estimated using analysis of variance (ANOVA). Favorable outcomes (i.e., less dependence in ADL, increased usability and self-rated health, and less concerns about falling) are marked with grey shading.

		Low Disability			Medium/High Disability			*p*-Value	
		*n*	M	SD	*n*	M	SD	Unadj	Adj
ADL	T1	69	9.5	5.5	34	14.4	6.3		
	T4	68	11.2	6.3	34	15.1	5.7		
	Change	68	1.7	4.9	34	0.7	3.4	0.284	0.940
P-ADL	T1	69	3.1	2.8	34	5.5	3.7		
	T4	69	3.8	3.5	34	5.7	4.0		
	Change	69	0.7	2.7	34	0.2	2.2	0.388	0.817
I-ADL	T1	69	6.4	3.2	34	9.0	3.1		
	T4	68	7.3	3.3	34	9.4	2.1		
	Change	68	1.0	2.9	34	0.5	2.4	0.389	0.676
Usability: Self-care	T1	69	20.6	3.9	34	12.1	5.8		
	T4	69	18.8	5.8	34	13.4	6.7		
	Change	69	−1.8	6.2	34	1.3	8.4	0.038	0.397
Usability: Social	T1	69	13.5	1.7	34	9.7	4.3		
	T4	69	12.8	2.6	34	10.8	4.0		
	Change	69	−0.7	3.0	34	1.1	6.4	0.054	0.244
Usability: Outdoor leisure	T1	69	9.7	3.0	34	4.8	3.1		
	T4	69	9.2	4.2	34	7.3	4.0		
	Change	69	−0.4	4.3	34	2.5	4.5	0.002	0.037
Concerns about falling	T1	57	15.0	4.8	26	18.6	6.2		
	T4	50	14.2	5.1	22	14.7	5.4		
	Change	43	−0.1	5.4	17	−3.6	7.7	0.045	0.100
Self-rated health	T1	69	61.4	18.4	30	48.8	20.4		
	T4	67	63.4	20.8	29	55.1	23.4		
	Change	67	1.9	23.7	26	3.1	24.9	0.830	0.324

Changes in outcomes are illustrated per client profile and aggregated profiles for ADL dependence (Figure 1), usability in the home (Figure 2), concerns about falling (Figure 3), and self-rated health (Figure 4). 

### 3.2. Differences in Changes in Outcomes between Client Profiles 

There were no statistically significant overall differences between the client profiles with respect to change in ADL, P-ADL, I-ADL, or self-rated health. For usability, there was a statistically significant difference in changes between the client profiles for self-care aspects and outdoor leisure aspects but not for social aspects. In the post hoc analyses, differences in changes were found between older adults with low level of disability and older adults with medium/high level of disability for self-care social and outdoor leisure aspects. 

When aggregating the profiles, differences were found in usability self-care aspects and social aspects as wells in in concerns about falling, but not in ADL or self-rated health.

When it comes to history of falls, older adults with medium/high level of disability were more than three times more likely than older adults with low level of disability to worsen over one year. None of the other profiles differed statistically significantly from older adults with low level of disability in risks of falls, but when comparing the aggregated profiles (low vs. medium/high disability), a three-fold risk of worsening of fall history were found for those with medium/high disability at baseline. There were no differences in changes in the fall history between the single profiles or between the aggregated profiles (Table 5). 

## 4. Discussion

This study investigated one-year changes in ADL, usability in the home, history of falls, concerns about falling, and self-rated health across different HA client profiles. It further investigated differences in changes over one-year between the profiles. The changes in the different client profiles demonstrate a complex pattern of improvements and declines, depending on outcome targeted, with no profile showing consistent improvements or decline across all outcomes. No consistent, significant differences between client profiles were found either. 

At baseline, older adults with low level of disability gave an overall impression of being quite vulnerable and at definite risk of declining physical function [23]. This is at least partially confirmed by the results in the current study. Given the close relationship between ADL and usability, the difference in changes in the two outcomes could be anticipated. Usability is explicitly related to the physical environment, and our results indicate that the home environment one year after the HA does not fit the needs of the client any longer. Older adults with medium/high level of disability had a considerable disability already at baseline but when it comes to ADL and usability of the home, they probably benefited from the HA also over such a long time period as one year. This group, however, reported increased concerns about falling and were at a statistically significantly higher risk of actually worsening in fall history compared with older adults with low level of disability. Most probably, people with these profiles are at risk of increased ADL dependence if their home environment is not further adapted to their needs [15]. This is also indicated by the increased concerns about falling clients with this profile report after one year. It is well-known that concerns about falling is a risk factor for actual falls due to individual behavior changes, directly contributing to increased falls by changes in gait patterns [32], or indirectly by leading to a vicious cycle of activity avoidance and functional decline. Ultimately, this may contribute to increased disability [33,34,35]. Thus, despite the improved self-rated health reported among these clients, it seems that they are at risk of increased disability if not evaluated regularly. 

Adults with low level of disability showed less dependence in ADL and considered the usability of their home better over one year. They seemed thus to benefit from the HA over the entire follow-up period. Due to their relatively young age, they were most probably also more able to adapt to changing physical and environmental conditions. However, at the same time they most probably expected and strived for being able to lead a life similar to most people in their own age. While experiencing health decline in old age can be considered an “on-time” event, naturally increasing with increasing age, in younger age disability is more unusual [36,37]. Comparisons with people of the same age may be one of the reasons for their decline in self-rated health over time. 

Adults with high level of disability, and older adults with medium level of disability including at least moderate cognitive impairment had cognitive impairments already at baseline, and their patterns of decline or no change over time were expected. People with cognitive impairments increasingly become dependent in ADL and other activities [38,39], and they, to a large extent, lack the capacity to adapt to functional decline and to changes in the physical environment [40]. They most often reduce the number of activities they perform or perform them with significant assistance from others. Thus, they become less aware of barriers in the environment that might affect activity negatively, i.e., they may assess the usability of their home based on previous activity, not current. That is, without constant monitoring of their everyday situation, people with cognitive impairments are at risk of rapid and considerably increased dependence in ADL and subsequent disability. Given the strong relationship between disability and old age, also seemingly healthy older people are at risk of rapidly increasing disability, due to their lack of reserve capacity to adapt to declining function or changes in their environment. More specifically, the less capacity (physical/cognitive) in the person, the less potential to adapt to further decline and the higher the need for adaptation of the environment to maintain activity [41]. 

In the aggregated profiles, usability of the home turned out to decline among those with low disability at baseline but not among those with medium/high disability. On the contrary, the risk of experiencing a worsening fall history one year after the HA was three times higher among those with medium/high disability compared with those with low, while the clients in this profile became less concerned about falling over time. People with cognitive impairments and people who are concerned about falling or already have fallen are at considerable risks for disability and needs careful monitoring to avoid additional falls and increasing disability. In this context, it is important to remember that the people included in this study had been granted a HA, i.e., already at baseline they had problems with being active and independent due to the physical environment in their own home. Thus, at baseline all participants could be considered at risk of functional decline even if their level of disability was low at the time of application for the HA. That is, applying for a HA is an indication that a person’s health trajectory is negative and needs careful monitoring. For people with declining function, even small changes in the environment, such as a HA, can have a major impact on their possibilities to lead an independent life [40]. Most importantly though, person–environment–activity transactions are complex, and due to different factors, the positive outcomes of a HA in terms of less ADL dependence and increased usability may not persist over a longer time. This explains the complex pattern of improvements and declines demonstrated by our findings. 

From a public health perspective, it has been shown that environmental barriers are considerably prevalent in the ordinary housing stock. These barriers will cause problems for older people, such as decline in usability, more dependence in ADL, and more concerns about falling, even if they seem to function extremely well [4,41]. Younger people with high levels of disability often have developed strategies to overcome barriers in the environments or decline in the own functions, at least if they do not have any cognitive impairments, but efforts to design the environment accessible, usable, and supportive can of course prevent disability also among them [4]. In fact, it has been suggested that effects of making a home more accessible in terms of postponed or reduced need for home help services, informal care, and special housing would be economically beneficial for the municipalities despite the costs of the reconstruction [42].

Within health care, all interventions should be appropriate, effective, cost-effective [43], and based on current research evidence [44]. Moreover, to come up with health services priorities [45] and for the purpose of evaluations of outcomes [42] as well as costs, more detailed knowledge about the population is required, for example their disability trajectory [46,47]. This kind of knowledge can be useful to determine follow-up schemes in practice and to implement individually tailored interventions to prevent further decline. Such interventions can target both the environment and the person, as well as the activity to be performed. 

Although there were consistent patterns in the results, for example, that people with low disability levels had larger changes in all ADL measures than people with medium or high disability levels, only a few statistically significant differences were found. One explanation may be the small sample size in each group, also in the aggregated profiles, and thereby a lack of power to detect real differences. Prior to the study, power analyses were conducted based on data on ADL dependence in a similar sample of HA clients, indicating a sample size large enough to detect differences [7]. However, as common in studies targeting older people and people with disabilities, in our study high attrition rates resulted in a lower sample size than desired. Measures were undertaken to reach a sufficient sample size, such as a substantial prolongation of the data collection period to include more study participants. The group of people receiving HA in Sweden is increasingly facing health decline, often quite rapidly, which explains the high number of dropouts over one year in our sample. This affects their possibilities to participate in research studies requiring time and energy. This is the case for people with cognitive decline, which constitute a considerable amount of those declining further participation in our study, but also due to other health conditions.

## 5. Conclusions

To conclude, the risks of deterioration over one year were the highest among those with cognitive impairments. However, with respect to client profiles, no clear indications were found regarding which profiles deteriorated or were at the highest risk of deterioration. The results of our study add valuable knowledge about the heterogeneity of HA clients and how different groups of clients change over time in different aspects of health. Profile membership can to some extent inform health care practitioners on the prospective trajectories of the clients after the HA implementation. However, our results were not clear-cut enough to recommend prioritization and monitoring decisions based on baseline client profiles. Instead, it seems that all HA clients, independently of baseline profile, are at risk of increasing functional decline and disability over time and require follow-up evaluations regularly. This kind of knowledge is useful for municipality health care practitioners and decision-makers on how to allocate resources to those in best need to counteract disability in the population. 

## Figures and Tables

**Table 1:** Characteristics of the five client profiles.

	Age	ADL Dependence	Functional Limitations	Cognitive Impairment	Concerns About Falling	Usability of the Home
Older adults with low level of disability	Old	Low	Low	No/mild	Low	High (all)
Older adults with medium/high level of disability	Old	Medium/High	Medium	Mild	Medium	Self-care: medium Social: medium Leisure: low/medium
Adults with low level of disability	Young	Low	Low	Mild/no	High	Self-care: high Social: high Leisure: low/medium
Adults of high level of disability	Young	High	High	Mild	High	Self-care: low Social: low Leisure: medium
Older adults with medium level of disability and cognitive impairment	Old	Medium	Medium/High	Moderate/Severe	Medium	Medium (all)

**Table 2:** Study population description, total sample, and profile per client.

		Total	Older Adults, Low Disability	Older Adults, Medium/High Disability	Adults, Low Disability	Adults, Medium/ High Disability	Cognitive Impairment
Age (years)	n	108	59	26	10	8	5
	M (SD)	75 (13)	79 (7)	80 (7)	49 (6)	54 (15)	78 (8)
Gender, *n* (%)	Male	29 (27)	11 (19)	10 (38)	3 (30)	3 (38)	2 (40)
	Female	79 (73)	48 (81)	16 (62)	7 (70)	5 (63)	3 (60)
Living situation, *n* (%)	Alone	66 (61)	41 (70)	13 (50)	5 (50)	4 (50)	3 (60)
	With someone	42 (39)	18 (31)	13 (50)	5 (50)	4 (50)	2 (40)

M = Mean; SD = Standard deviation.

**Table 5:** Odds ratios (ORs) with 95% confidence intervals (CIs) for worsening of fall history during one year after HA.

	Worsening				Unadjusted OR (95% CI)	Adjusted OR (95% CI)
	No		Yes			
	*n*	%	*n*	%		
**Original client profile**						
Older adults, low disability	35	69	16	31	1.00 (ref)	1.00 (ref)
Older adults, medium/high disability	7	39	11	61	**3.44 (1.13–10.5)**	**3.83 (1.02–14.3)**
Adults, low disability	7	70	3	30	0.94 (0.21–4.10)	1.22 (0.24–6.10)
Adults, medium/high disability	3	60	2	40	1.46 (0.22–9.60)	9.54 (0.43–209)
Cognitive impairment	2	67	1	33	1.09 (0.09–13.0)	1.40 (0.06–32.0)
**Aggregated client profiles**						
Low disability	42	69	19	31	1.00 (ref)	1.00 (ref)
Medium/high disability	10	43	13	57	**2.87 (1.07–7.71)**	**3.60 (1.01–12.8)**

Statistically significant figures are presented in bold.

## Data Availability

The data presented in this study are available on reasonable request from the corresponding author. The data are not publicly available due to participant confidentiality.
